# Salt stains from evaporating droplets

**DOI:** 10.1038/srep10335

**Published:** 2015-05-27

**Authors:** Noushine Shahidzadeh, Marthe F. L. Schut, Julie Desarnaud, Marc Prat, Daniel Bonn

**Affiliations:** 1 Van der Waals-Zeeman Institute, Institute of Physics, University of Amsterdam, Science Park 904, 1098 XH Amsterdam, The Netherlands; 2 Université de Toulouse, INPT, UPS, IMFT and CNRS, Toulouse, France

## Abstract

The study of the behavior of sessile droplets on solid substrates is not only associated with common everyday phenomena, such as the coffee stain effect, limescale deposits on our bathroom walls , but also very important in many applications such as purification of pharmaceuticals, de-icing of airplanes, inkjet printing and coating applications. In many of these processes, a phase change happens within the drop because of solvent evaporation, temperature changes or chemical reactions, which consequently lead to liquid to solid transitions in the droplets. Here we show that crystallization patterns of evaporating of water drops containing dissolved salts are different from the stains reported for evaporating colloidal suspensions. This happens because during the solvent evaporation, the salts crystallize and grow during the drying. Our results show that the patterns of the resulting salt crystal stains are mainly governed by wetting properties of the emerging crystal as well as the pathway of nucleation and growth, and are independent of the evaporation rate and thermal conductivity of the substrates.

Water evaporation appears in many guises throughout everyday life; we are very familiar with salt deposits such as limescale in our kettle or on our bathroom walls. Although evaporation as a bulk phase transformation is well understood, the evaporation of droplets is an active area of research. Especially when solutes are present, new phenomena and a rich variety of possible behaviors can be obtained: the “coffee-stain phenomenon”^1^,^2^,^3^ and its reversal^4^,^5^,^6^,^7^, have been the subject of intense study as the process can be observed for many types of suspended materials and is of major importance for various applications such as inkjet printing, coating applications and the manufacturing of novel electronic and optical materials^8^.On the other hand, in the pharmaceutical industry the formation of crystals with controlled size, shape, purity and polymorphism is critical^9^. The evaporation is fastest near the edge of an evaporating drop, and consequently when the contact line is pinned at the surface, the solvent lost by evaporation around the edge must be replenished; this causes a flow in the droplet that brings fluid from the center to the edge of the droplet carrying with it any solutes which are then deposited at the edge, forming a ring-like stain^1^,^2^. By sequences of pinning and depinning one can even obtain multiple rings^3^ or regular patterns^2^. However this does not always happen; for very volatile solvents, it was reported that the coffee-stain effect can be inversed by thermal Marangoni flows^6^. Marangoni flows arise due to surface gradients along the interface because of the non-uniform evaporation, which draws energy unevenly from the drop creating a temperature gradient. Consequently, the thermal conductivity of the substrate can even change the direction of the Marangoni flow and the resulting deposition pattern^7^. Although the deposition pattern has been much studied for evaporating droplets of colloidal suspensions, very few studies exist of evaporating salt solutions , in spite of the fact that water usually contains dissolved salts, and that the much-studied colloidal systems are usually considered to be models for atomic systems^10^. The evaporation of sessile drops of electrolyte solutions is commonly used to study the precipitation mechanisms but little attention has been paid to the resulting patterns^11^,^12^,^13^,^14^.

We therefore investigate the crystallization and the deposit patterns of aqueous solutions of two different salts during evaporation. To assess possible Marangoni effects, surfaces of different wettability and thermal conductivity are considered. As salts, we use Sodium Chloride (NaCl) and Calcium Sulfate (CaSO_4_); these salts are selected firstly because of their importance in many natural and industrial processes^15^,^16^ and secondly because they have different crystalline structures and precipitation pathways^17^,^18^,^19^,^20^,^21^. Sodium chloride (NaCl) forms in general cubic anhydrous crystals. Calcium sulfate, on the other hand, has three crystalline polymorphs that differ in their degree of hydration: anhydrite (CaSO_4_), hemihydrate (“bassanite”: CaSO_4_, 0.5H_2_O), and dihydrate (“gypsum”: CaSO_4_, 2H_2_O). The precipitation of gypsum, the stable polymorph under ambient conditions, is of much current interest since it has been shown to pass through non-classical nucleation pathways via amorphous and hemihydrate intermediates^19^,^20^.

## Results

**Figure 1:**
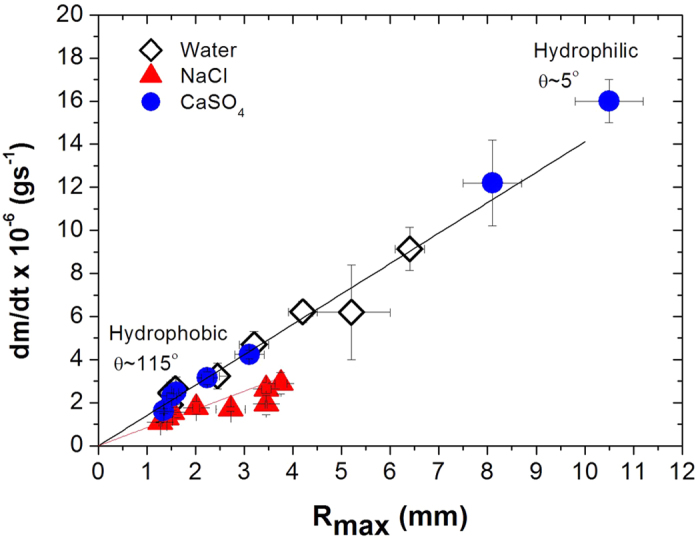
Evaporation rates (J = dm/dt) as a function of the maximum radius of the deposited droplets (V_0_=10 μl) on different substrates (glass, silicon wafer, PDMS) with different wetting properties (see Table 1). The latter increases linearly with drop radius (J = aR + b with b = 0 , a_CaSO4_ = 1.41 10^−6^, a_NaCl_ = 8.45 10^−7^). Relative humidity RH ∼ 50 ± 2%, temperature T ∼ 22 ± 2 °C. squares: water; triangles: NaCl droplets (m_i_ = 5.9 mol kg^−1^); circles: CaSO_4_ droplets (m_i_ = 0.014 mol kg^−1^).

We study the evaporation rate and resulting crystallization patterns for aqueous drops on a variety of different surfaces with different wetting properties and thermal conductivities; the different systems are detailed in the Methods section. Measuring the evaporation rates of 10 μl droplets of water, NaCl and CaSO_4_ solutions for different contact radii R_max_ of the deposited droplets show that, independently of the type of solid surface, the evaporation rate always increases linearly with the drop radius (Fig. 1). Thus, the evaporation rate dV/dt is proportional to the perimeter of the droplet dV/dt ∼ 2π R (as expected^1^,^2^,^22^), implying that a drop of a given volume has a slower evaporation rate on a hydrophobic surface compared to the same drop on a hydrophilic surface due to a smaller radius in the former case. Comparing different surfaces of different thermal conductivities, it is clear that the conductivity does not influence the evaporation rate significantly: the evaporation is not limited by heat transfer from the surface into the drop. On the other hand the wetting properties do influence the evaporation rate by changing the radius of the droplet.

It can also be observed in Fig. 1 that CaSO_4_—containing droplets have higher evaporation rates in comparison to NaCl-containing droplets for a given surface. This is mainly due to two effects: first, the saturated water vapor pressures over aqueous salt solutions are different because of the much higher solubility of NaCl: in terms of the equilibrium relative humidity above the salt solutions 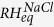 ∼ 75% while 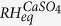 ∼ 100%. Second because the liquid-vapor surface tension of NaCl solutions is significantly above that of water, drops of NaCl and CaSO_4_ solutions have different contact angles on a given substrate (Table 1). In fact, the physicochemical properties of CaSO_4_ droplets being very similar to those of pure water lead to an very similar evaporation rate as for pure water.

**Figure 2:**
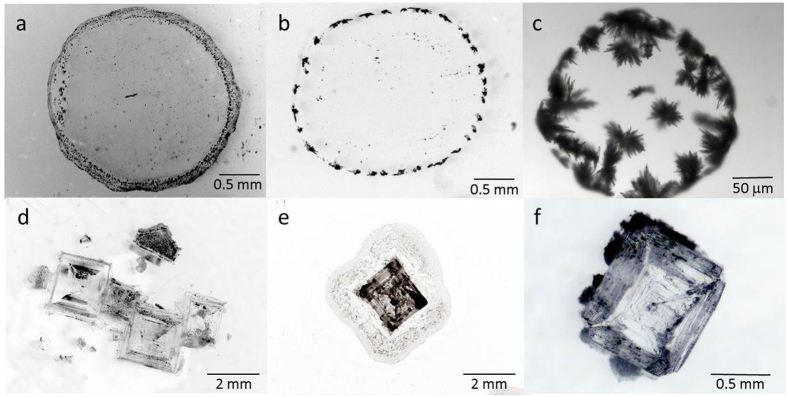
Crystallization pattern of two types of salt solution droplets at the end of drying on three types of glass slides with different wetting properties. Top panels (a,b,c): CaSO_4_; bottom panels (d,e,f): NaCl. Left panels (a,d): Piranha cleaned; middle panels (b,e): Ehtanol+ Millipore water cleaned; right panels (c,f): silanized.

**Figure 3:**
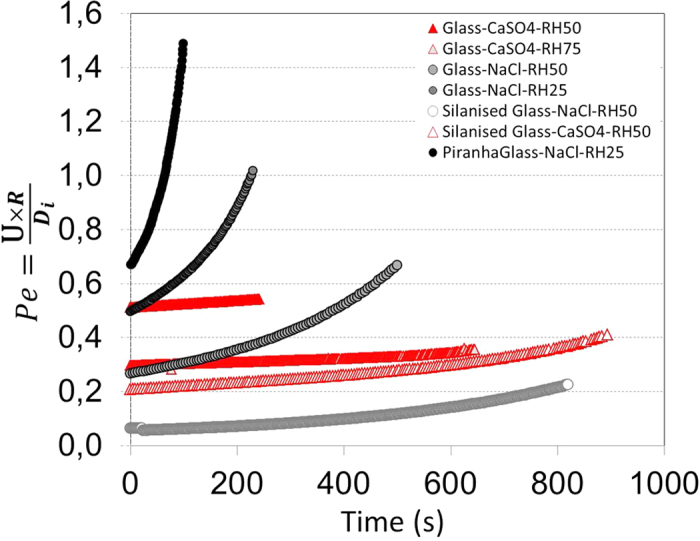
Peclet numbers calculated for droplets of 1 μl size on cleaned glass slides (ethanol +water and Piranha) and silanized glass for the two different evaporation rates (see methods) till the precipitation of crystals in droplets.

During the drying, the salt solutions in the drops become supersaturated, and subsequently the salts will crystallize. The crystal deposits left after complete drying for both salts on various solid substrates show that the final crystallization patterns are very different for the two salts (Fig. 2). Systematically, CaSO_4_ crystallization leads to a ring-like deposit with a large number of crystals whereas NaCl crystals show no coffee-stain effect at all. In addition, for a given salt , the crystal patterns are independent of the size of the droplet as well as the thermal conductivity and the wetting properties of the substrates (Fig 2; see also Fig S1 & S2 in supplementary information). In order to see if this difference is not related to the different evaporation rates between the two salts, ((dV/dt)_NaCl_ ∼ 710^−13^ m^3^ s^−1^) and (dV/dt)_CaSO4_ ∼ 1,910^−12^ m^3^ s^−1^) for droplets V_0_ ∼ 1 μl), we also performed experiments in which the relative humidity was chosen such that the same evaporation rate was obtained for the two salt solutions: a relative humidity of 25% for NaCl and 75% for CaSO_4_ containing droplets achieve this. The result shows that the morphology of the stains are not affected by the evaporation rate. For the two evaporation rates, we have estimated the ratio of the rate of advection of ions by the flow to the rate of diffusion in the salt solution droplets by calculating the Peclet number,: Pe=RU/D_i_ where R is the radius of the droplet, U the flow velocity in the drop and D_i_ the diffusion coefficient of the ions in water (Fig. 3). For each experiment, the typical flow velocity U in the drop was estimated from the total flux J = −dV/dt and the area of the spherical cap *A(t)* of the drop. For CaSO_4_ solutions, the diffusion coefficient of the Ca^2+^ ion D_Ca_^2+^ = 0,79210^−9^ m^2^/s^22^ is constant since the solubility remains very low (m_sat_ = 0.015 mol. kg^−1^) while for for Na^+^ D_Na+_ = −0,1097 10^−9^ *m(t) *+ 1,324 10^−9^ with *m*(t) the concentration in (mol. kg^−1^) in the drop at the time t since the concentration increases up to ∼10 M during evaporation before crystals precipitate ;the increase in concentration changes the viscosity of the solution, which affects the diffusion coefficient^23^,^24^. We find that in all of the CaSO_4_ experiments, Pe <1 (Fig. 2), implying that diffusion dominates over advection, and the solution remains homogeneous in concentration. For fast evaporation of NaCl-containing droplets (at RH ∼ 25%), Pe can exceed unity before crystallization for very hydrophilic substrates, However, our results show that the fast evaporation induces the precipitation of a larger number of crystals, presumably due to the heterogeneous distribution of ions. However, even though convection dominates over diffusion, we observe no ring-like deposits at the end of drying (Fig. 2b). The insensitivity of the results to changes in Pe also shows up when we increase the contact angle from hydrophilic to hydrophobic at given relative humidity (RH ∼ 50%). For the hydrophobic surface again the evaporation is slower, but the lower values of Pe do not result in a change in the resulting salt stains; this holds for either of the salts.

**Figure 4:**
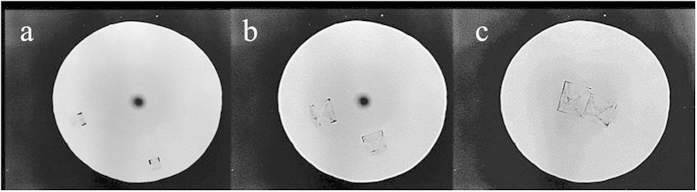
Phase contrast microscopy images of the inward movement of NaCl crystals during evaporation of a NaCl solution droplet (V_0_ = 0.2 μl) on glass slide.

**Figure 5:**
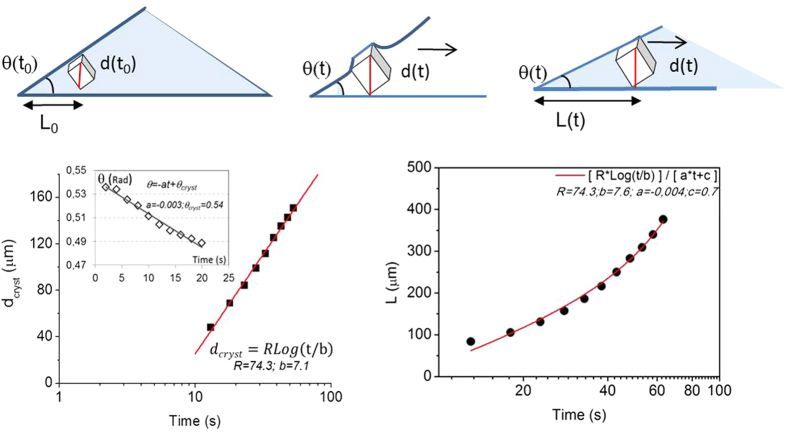
Top: schematic presentation of the inward movement of the crystal during its growth due to capillary forces. **left:** The growth of NaCl crystals during droplet evaporation as a function of time. The inset shows the time evolution of the contact angle θ (t) of NaCl droplet on a glass substrate after the crystallization; **right:** The inward displacement L(t) of one crystal due to the lateral capillary force; the curve is given by the geometrical constraint *L(t)* = *d(t)/θ(t)* and perfectly describes the inward motion of the crystal.

We conclude that, the two different morphologies obtained for the two salts (coffee stain for the CaSO_4_ and no coffee stain for the NaCl) cannot be the effect of a Marangoni flow for NaCl (as reported for the anti-coffee stain effect^6^) since it happens for both slow and fast evaporation experiments and on both thermally conducting and isolating surfaces. To assess where the difference between the two salts comes from, we have studied the kinetics of nucleation and growth during evaporation of the drops on different substrates. For NaCl droplets, we observe that the first crystal is formed at the liquid/air interface^11^ close to the contact line. From the calculation of Pe, we surmise that the nucleation close to the edge is likely due to a higher concentration of the solute near the edge of the droplet. The maximum supersaturation achieved at the onset of nucleation and growth is S = *m*/*m*_*sat*_ ∼ 1.6 ± 0.2 in good agreement with previous results (figure S3 in supplementary information)^17^. Surprisingly, during its growth, the cubic crystal shows an inward motion towards the center of the droplet (Fig. 4). When the crystal size increases, the crystal becomes confined between the free surface and the solid substrate. The resulting deformation of the liquid/air interface due to this geometrical constraint results in capillary forces that push the crystal towards the center of the drop^4^. Quantitatively, the displacement of the crystal from the edge towards the center *L (t)* can be described by considering that a cubic crystal of size *d(t)* fits in the corner of a drop with contact angle θ(t) when the distance between the edge and the crystal satisfies *L(t)* *= d(t)/tan (*θ). By independently measuring the contact angle θ(t) and the crystal growth d (t) during evaporation, we find that θ(t) linearly decreases once crystallization starts, and the crystal size itself *d(t)* is logarithmically increasing in time (Fig. 5).

The latter behavior is due to the rapid drop of the salt concentration due to the growth of the crystal. It is well known that for a constant supersaturation crystal growth is linear in time^25^; the slower growth thus implies that the growing crystal consumes ions from the solution and that consequently the supersaturation is decreasing; more quantitative calculations of this effect can be found in^26^,^27^.

For NaCl containing drops, the combined effects of the increase in crystal size and the decrease of the contact angle quantitatively explain the measured inward motion of the crystal , as can be observed in Fig. 5; this motion prevails over the usual outward flow of ions causing coffee stains and leads to the unexpected deposit pattern at the end of drying. On the other hand, for the CaSO_4_ containing droplets, the crystallization starts at the solid/liquid interface near the contact line of the droplet and this then leads to the coffee-stain like patterns at the end of drying, (Fig. 2).

The observed coffee stains for CaSO_4_, however are much richer than just a single crystalline species. We find that the wetting properties of the substrate directly control the degree of confinement of the solution near the contact line, which in turn determines which polymorph precipitates as well as its orientation. The type of crystal formed is observed to depend directly on the contact angle of the drop; four different cases, corresponding to four different morphologies of the final patterns are observed.

**Figure 6:**
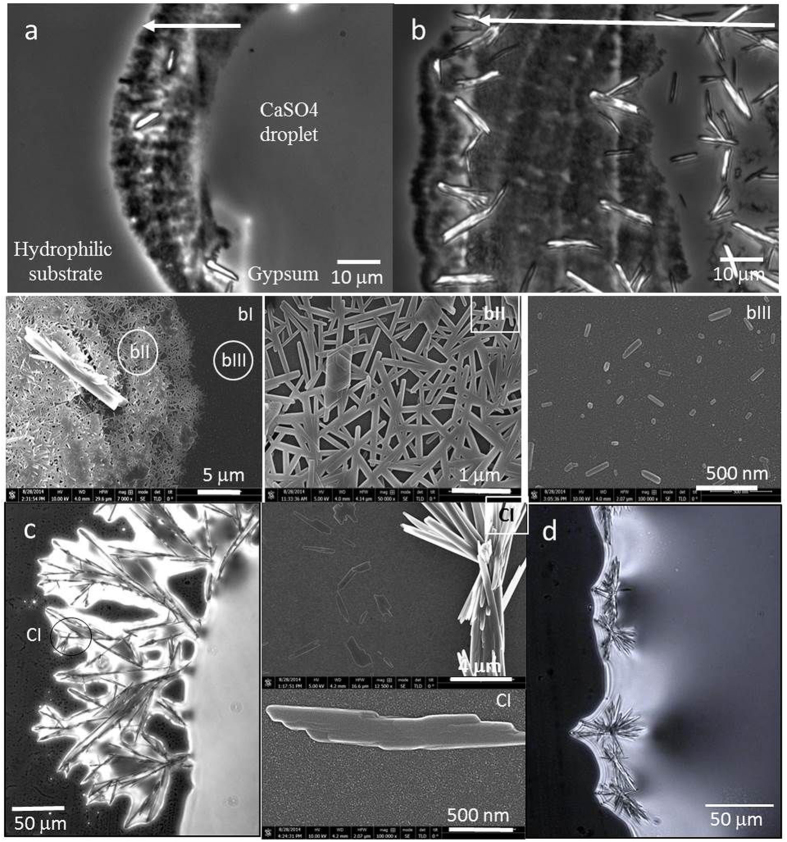
Phase-contrast and High resolution SEM micrographs of the growth of CaSO_4_ polymorphs for various contact angles *θ* of the droplet with the glass substrate. **(a-b)** θ ∼ 5°: formation of an intermediate grey phase at the contact line followed by the spreading of the droplet with time (time between a and b images ∼5 s). The arrow points are the spreading region from right to left. (bI-III) High-resolution SEM images of CaSO_4_ crystals at the contact line of the droplet. Presence of nanorods and nanoparticles of bassanite and growth of gypsum from their assembly (Mikado-like structure) **(c)** θ ∼ 13°: direct formation of gypsum needles in the bulk at the contact line region and spreading of the solution around the needles.(CI) High-resolution SEM images of CaSO_4_ crystals at the contact line region, presence of nanocrystals of Gypsum **(d)** θ ∼ 30°. clusters of interpenetrating gypsum needles (“sea urchin” shape) without the spreading of the droplet.

CASE1. For very low contact angles (θ ∼ 5°), the crystallization of gypsum needles is observed via the formation of an intermediate phase (grey zone in Fig. 6a,b) at the edge of the droplet. High-resolution electron microscopy of this zone confirms the presence of a different polymorph of CaSO_4_: we observe nanorods and nanoparticles of bassanite^19^,^20^ that have aggregated prior to the formation of gypsum (Fig. 6 bI-III). Interestingly, the formation of these small nano crystals appears to originate from the precursor film that extends far from the macroscopic drop (fig. 6-bIII).

CASE 2. For a slightly larger contact angle (θ ∼ 15°) which gives a less confined solution at the contact line, the gypsum crystal needles grow directly from the solution; no nanorods or nanoparticles of bassanite are observed prior to the precipitation of gypsum (fig. 6c). The Gypsum needles cluster together due to capillary forces^28^ to form a Mikado-like structure.

For both cases discussed above with contact angles θ < 30°, the deposition of small cristallites on the solid surface at the edge of the drop is observed to enhance the spreading of the droplet around these crystals (Fig. 4a,b and c), leading to the formation of crystals well beyond the initial contact radius. This can be viewed as a consequence of the fact that roughness on surface makes a hydrophilic substrate more wettable^29^. The formation of crystals on the surface makes the surface more wettable to the solution and consequently, a new advancing contact line is created by the advancing liquid. This in turn leads new crystals that will precipitate away from the original drop edge resulting in a ‘halo’ of small crystals around the coffee stain formed by the original drop.(Figs. 6 and 2a). The smaller the crystal size (i.e. the lower the contact angle for the CaSO_4_ solution), the broader and the more extended the ‘halo’ becomes. This is generally known as çreeping motion of salts, a phenomenon that remains ill understood^31^,^32^,^33^; our results show the direct impact of the wettability and the interfacial properties of the emerging crystal on creeping.

CASE 3. For larger contact angles (θ > 30°) , the crystals grow as clusters of interpenetrating needles (in a ‘sea urchin’ shape), which corresponds precisely to the bulk solution morphology of gypsum^19^,^30^. These sea urchins nucleate and grow onto the solid substrate and are anchored to it; Fig. 6d shows that they are capable of deforming the liquid-vapor interface.

All of these results for CaSO_4_ for different contact angles are in very good agreement with previous experiments on the effect of confinement on the CaSO_4_ precipitation from solution^30^. Therefore, evaporating droplets on substrates with different wetting properties could be used as a simple way of studying different polymorphs for crystals which precipitate in the solid/liquid interface.

**Figure 7:**
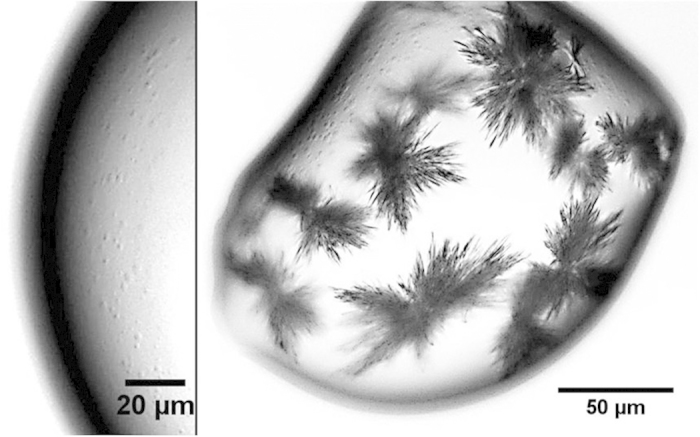
Phase-contrast micrographs of the evaporation of CaSO_4_ droplets on hydrophobic substrates (silanized glass and PDMS). Left: mesoscopic clusters (dense liquid precursors) at supersaturation S ∼ 6 ± 0.2, just before gypsum growth. Right: rapid growth of gypsum crystals and remaining mesoscopic clusters.

CASE 4. For the largest contact angles contact angle (θ > 90°), the effect of confinement becomes negligible and a homogeneous distribution of ions through the droplet is achieved due to the low Pe number that results from the slow evaporation. Here, the situation is thus comparable to bulk nucleation from a homogeneous solution. In this case, we find an extremely high supersaturation at the onset of gypsum crystallization: S = m_c_/m_0_ ∼ 6, with *m*_*c*_ and *m*_*0*_ the molalities at the onset of crystallization and at the equilibrium respectively. At these high supersaturations, the surprising observation is that the nucleation and growth occurs via a large number of metastable mesoscopic clusters (‘dense liquid droplets’, Fig. 7). These clusters are spherical (and hence we expect them to be liquid rather than crystalline). They are observed to form at extremely high supersaturation and to attain a roughly constant radius on the order of a few micrometers; their lifetime is rather short (∼1-2 s) after which they transform into gypsum crystallites. Their short lifetimes makes them difficult to study systematically, but the fact that they arrive reproducibly at very high supersaturations suggest that the solution becomes unstable and forms these clusters that then transform into many crystallites. It is now well established that similar non-classical nucleation processes via stable prenucleation clusters happens for biominerals^34^ and proteins^35^,^36^. However, to our knowledge a direct observation of such large mesoscopic clusters has so far not been reported for salts.

In summary, we have performed the study of the evaporation of droplets of two different salt solutions in different substrates. We find that the evaporation at the edge of the droplets drives a capillary flow in the liquid which consequently increases the probability of nucleation and growth close to the contact line . However, the final salt deposition pattern is mainly controlled by two parameters. First, the interfacial properties of the emerging crystal determine whether the crystallization happens at the solid/liquid , at the liquid/vapor or in the bulk. If the crystal forms at the liquid-air interface (as is the case for NaCl), it may be transported away from the contact line during its growth by capillary forces. Such an effect is not seen for CaSO_4_ crystals which are formed on the substrate and remained anchored there .The second important parameter is the amount of crystals that are formed. For NaCl, reproducibly only one or a few cystals form, and with such a small number it is difficult to form a coffee stain, even if they would remain near the contact line. For CaSO_4_, the non classical nucleation pathway taken by the salt seems to induce a large amount of clusters that subsequently transform into a large number of crystals. The precipitation of the large amount of crystallites in addition to their interfacial properties then leads to a coffee stain pattern.

## Methods

Homogeneous salt solutions, i.e. without crystal seeds, were prepared by dissolving NaCl and anhydrous CaSO_4_ (Sigma Aldrich grade >99%) in Millipore water (ρ ∼18.2 MΩ·.cm) at concentrations slightly under saturation; i.e. relative supersaturation S = *m*_*i*_*/m*_*0*_ ∼ 0.93 ± 0.02 where *m*_*0*_ represents the saturation concentration (*m*_*0NaCl*_ = 6.1 mol.kg^−1^, *m*_*0CaSO4*_ = 1.5 10^−2^ mol kg^−1^) and *m*_*i*_ the initial concentration.

The solutions were used 24 hours after preparation. The surface tensions of the different solutions and their contact angles on different substrates were measured using a Krüss Instrument based on imaging analysis (Table 1). The contact angle of a liquid droplet is mainly dependent on the wettability of the solid substrate following Young’s equation which gives the relation between the equilibrium contact angle, which the drop makes with the surface, and the three surface tensions: 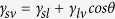
38.

We have performed evaporation experiments on droplets with volumes V_0_ of 0.2 *μ*l, 1-2 *μ*l and 10 *μ*l; For small droplets (1-2 *μ*l) processing of the data allows to determine the volume V, the surface area A, the base radius R, the contact angle θ and the height h and the concentration of the solution throughout drying till crystals start to grow in the solution. For droplets of 10  μl, the weight and radius of deposited droplets are monitored as a function of time on an automated balance with a precision of ±0.0001 g.

The evaporation of droplets was done in controlled climatic chamber at T = 22 ± 1 °C and relative humidities. Details on how the relative humidity is controlled in our climatic chamber can be find in^26^. Note that at a given relative humidity, the evaporation rates are different for the sodium chloride and calcium sulfate solutions because of the different water vapor pressures over aqueous salt solutions , and also their different contact angles in a given substrate (see table 1). At RH=50%, (dV/dt)_NaCl_ ∼ 710^−13^ m^3^ s^−1^ ) and (dV/dt)_CaSO4_ ∼ 1,910^−12^ m^3^ s^−1^) for droplets V_0_ ∼ 1 μl. Consequently, we also performed experiments at the same evaporation rate, we have adjusted the evaporation rate of one salt to the other by tuning the relative humidity of the climatic chamber to RH ∼ 75% (for CaSO_4_) and to RH ∼ 25% (for NaCl). At the end of the drying process the crystal deposit is analyzed. In parallel, the kinetics of nucleation and growth of crystals during evaporation of the droplets is followed at microscale using phase-contrast microscopy and a miniature climatic chamber^26^ . The identification of the crystalline phases at nanoscale were done using a High resolution Scanning Electron Microscope (VERIOS 460- Immersion mode) combined with an EDS (Oxford X-Max80 T) for elemental analysis.

The effect of thermal conductivities on evaporation and crystallization were studied by using different solid surfaces: PDMS (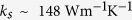), glass slides (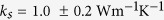) and Silicon wafers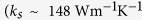)^24^. Seeing that each substrate has different wetting properties and in order to be able to compare the results on different substrates, the wettability of the surfaces were controlled by different types of treatments. Hydrophobic surfaces were prepared by silanization (Dynasylane OCTEO from Evonik)^39^ and hydrophilic surfaces either by air plasma treatment (treatment time 2 min) for PDMS and Silicon wafers or by the Pirhana cleaning process for glass slides. For the latter, different contact angles for hydrophilic surfaces were obtained by drying the cleaned glass slides at room temperature or in a 100 °C oven for one hour.

## Additional Information

**How to cite this article**: Shahidzadeh, N. *et al*. Salt stains from evaporating droplets. *Sci. Rep*. **5**, 10335; doi: 10.1038/srep10335 (2015).

## Supplementary Material

Supplementary Information

## Figures and Tables

**Table 1:** Contact angles of water, NaCl and CaSO_4_ droplets on PDMS, glass slide and Silicon wafer surfaces with different treatments: silanization with Dynasylan Octeo (EVONIK), air plasma treatment (2 minutes), cleaning with a Piranha solution followed by drying at room temperature or in the oven (100 °C) for one hour.

	Water	CaSO_4_ *m*_*i*_ *= 0.014 mol.kg*^*−1*^	NaCl *m*_*i*_*=5.9* *mol.kg*^*−1*^
Surface tension (± 0.25 mN/m)	71.7	71.6	81.1
	**Contact angle**	***θ***_**c**_ **(±3°)**	
**Glass / Pirhana cleaned**	5°	5	23
**Glass/ Pirhana cleaned+oven**	8°	14	33
**Glass/Ethanol+water cleaned**	26	28	39
**Glass/ Silanized**	104	105°	106°
**PDMS/air plasma**	∼5°	∼8	20°
**PDMS**	112°	116°	116°
**Si/air Plasma**	∼5°	6°	20°
**Si**	40°	42°	50°
**Si /Silanized**	93°	94°	97°
