# Supplementary material for: Salt stains from evaporating droplets
Source: Sci Rep. 2015 May 27;5:10335. doi: 10.1038/srep10335 (PMC4445064; doi:10.1038/srep10335)
Supplement: Supplementary Information [file srep10335-s1.pdf]

Salt stains from evaporating droplets

Noushine Shahidzadeh [a], Marthe F.L. Schut[a], Julie Desarnaud[a], Marc Prat[b], and Daniel Bonn[a]*

Supplementary Information

The final crystallization patterns obtained on Silicon wafers and PDMS with different wetting properties are shown in figurse S1 and S2.

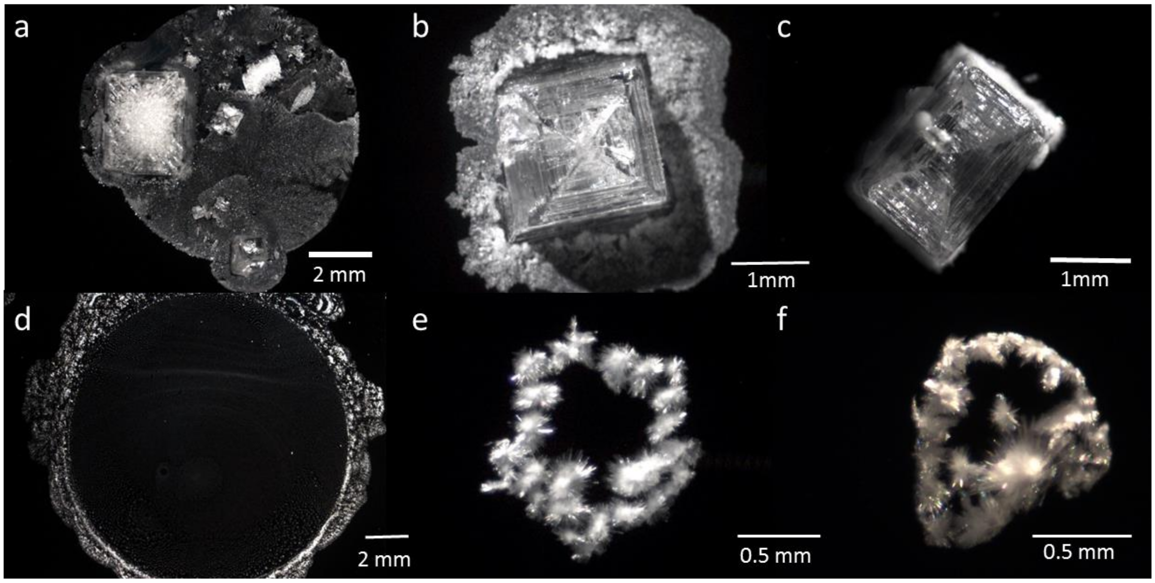

Figure S1. Crystallization pattern of two types of salt solution droplets at the end of drying on three types of silicon wafers with different wetting properties. Top panels (a,b,c): NaCl; bottom panels (d,e,f): $CaSO_4$. Left panels (a,d): air plasma treated; middle panels (b,e):untreated ; right panels (c,f): silanized.

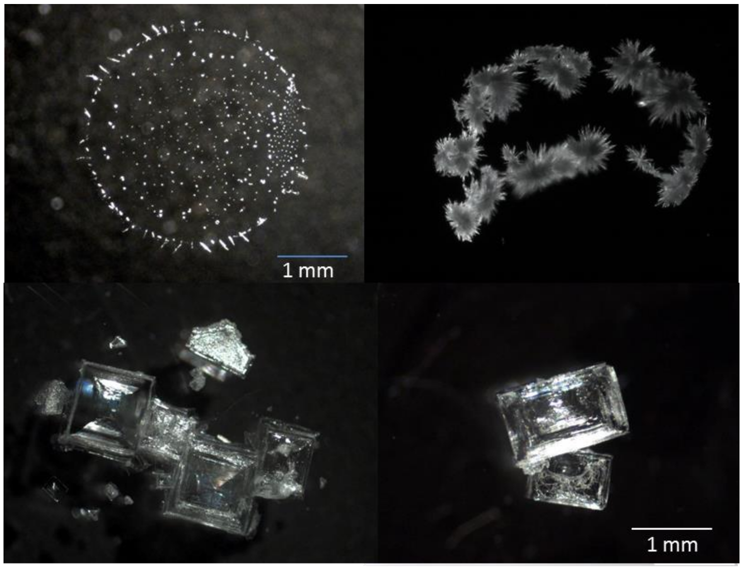

Figure S2. Crystallization pattern on PDMS . Top panels (a,b): $CaSO_4$; bottom panels (d,e): NaCl. Left panels: hydrophilic (air plasma treated); right panels: hydrophobic (untreated).

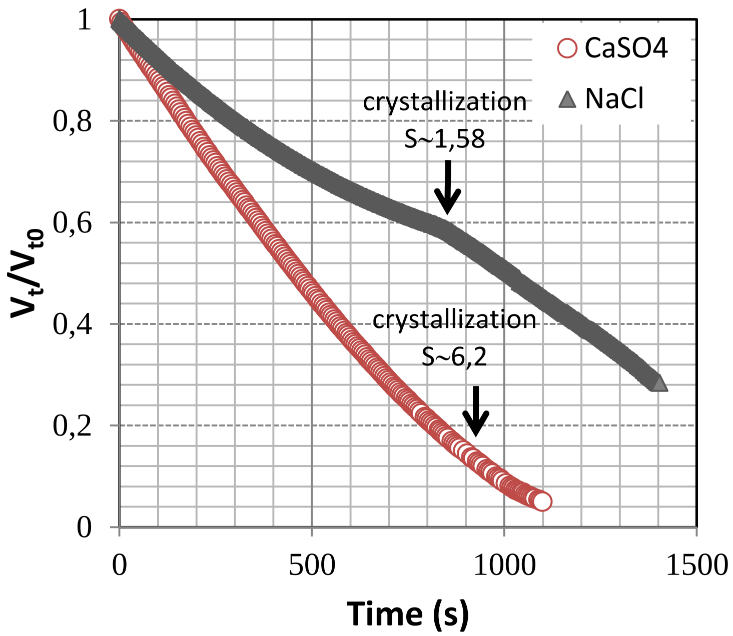

Figure S3. Normalized volume variation of aqueous $CaSO_4$ and NaCl droplets (V_0~1μl) on a silanized glass substrate at RH=50% and T=22°C. The supersaturations at the onset of

crystallization (S=m_{cryst}/m_0) are shown as the errows, and are deduced by simultaneously following the evaporation and the dynamics of growth under the microscope. In the case of sodium chloride the crystallization clearly changes the evaporation rate behavior due to the consumption of ions from the solution and the concomitant change in the equilibrium water vapor pressure above the solution [17].
